# Alcohol-attributed disease burden and formal alcohol policies in the Nordic countries (1990–2019): an analysis using the Global Burden of Disease Study 2019

**DOI:** 10.1093/eurpub/ckae195

**Published:** 2024-12-02

**Authors:** Lode van der Velde, Ahmed Nabil Shabaan, Anastasia Månsson, Peter Wennberg, Peter Allebeck, Thomas G Karlsson, Pär Flodin, Terje Andreas Eikemo, Ann Kristin Skrindo Knudsen, Indra de Soysa, Jens Christoffer Skogen, GBD 2019 Nordic Alcohol Collaborators, Hassan Abolhassani, Johan Ärnlöv, Ashokan Arumugam, Muhammad Asaduzzaman, Jennifer L Baker, Koustuv Dalal, Keshab Deuba, David Edvardsson, Seyed-Mohammad Fereshtehnejad, Rasmus J Havmöller, Simon I Hay, Knud Juel, Joonas H Kauppila, Ali Kiadaliri, Adnan Kisa, Sezer Kisa, Mika Kivimäki, Tea Lallukka, Anders O Larsson, Mall Leinsalu, Atte Meretoja, Tuomo J Meretoja, Junmei Miao Jonasson, Christopher J L Murray, Subas Neupane, Che Henry Ngwa, Gavin Pereira, Dominic Sagoe, Rahman Shiri, Thomas Clement Truelsen, Tommi Juhani Vasankari, Mika Gissler, John J McGrath, Inga Dora Sigfusdottir, Rannveig Sigurvinsdottir, Maja Pasovic, Anna-Karin Danielsson, Emilie E Agardh

**Affiliations:** Department of Global Public Health, Karolinska Institutet, Stockholm, Sweden; Department of Global Public Health, Karolinska Institutet, Stockholm, Sweden; Department of Global Public Health, Karolinska Institutet, Stockholm, Sweden; Department of Global Public Health, Karolinska Institutet, Stockholm, Sweden; Department of Public Health Sciences, Stockholm University, Stockholm, Sweden; Department of Global Public Health, Karolinska Institutet, Stockholm, Sweden; Public Health and Welfare, Finnish Institute for Health and Welfare, Helsinki, Finland; Department of Global Public Health, Karolinska Institutet, Stockholm, Sweden; Centre for Global Health Inequalities Research (CHAIN), Norwegian University of Science and Technology, Trondheim, Norway; Centre for Disease Burden, Norwegian Institute of Public Health, Bergen, Norway; Centre for Global Health Inequalities Research (CHAIN), Norwegian University of Science and Technology, Trondheim, Norway; Department of Health Promotion, Norwegian Institute of Public Health, Bergen, Norway; Alcohol and Drug Research Western Norway, Stavanger University Hospital, Stavanger, Norway; Region Stockholm, Academic Primary Health Care Centre, Stockholm, Sweden; Department of Molecular Medicine and Surgery, Karolinska Institutet, Stockholm; Knowledge Brokers, Finnish Institute for Health and Welfare, Helsinki, Finland; Queensland Brain Institute, The University of Queensland, Brisbane, QLD, Australia; National Centre for Register-based Research, Aarhus University, Aarhus, Denmark; Queensland Centre for Mental Health Research, The Park Centre for Mental Health, Wacol, QLD, Australia; Department of Psychology, Reykjavik University, Reykjavik, Iceland; Department of Health and Behavior Studies, Columbia University, New York, NY, United States; Department of Psychology, Reykjavik University, Reykjavik, Iceland; Institute for Health Metrics and Evaluation, University of Washington, Seattle, WA, United States; Department of Global Public Health, Karolinska Institutet, Stockholm, Sweden; Department of Global Public Health, Karolinska Institutet, Stockholm, Sweden

## Abstract

It is still unclear how changes in alcohol control policies may have contributed to changes in overall levels of alcohol-attributed harm between and within the Nordic countries. We modified and applied the Bridging the Gap (BtG)-scale to measure the restrictiveness of a set of alcohol control policies for each Nordic country and each year between 1990 and 2019. Alcohol-attributed harm was measured as total and sex-specific alcohol-attributed disease burden by age-standardized years of life losts (YLLs), years lived with disability (YLDs), and disability-adjusted life-years (DALYs) per 100 000 population from the Global Burden of Disease Study (GBD). Longitudinal cross-country comparisons with random effects regression analysis were employed to explore associations, within and across countries, differentiated by sex and the time to first effect. Overall, alcohol-attributed YLLs, YLDs, and DALYs decreased over the study period in all countries, except in Iceland. The burden was lower in those countries with restrictive national policies, apart from Finland, and higher in Denmark which had the least restrictive policies. Changes in restrictiveness were negatively associated with DALYs for causes with a longer time to effect, although this effect was stronger for males and varied between countries. The low alcohol attributed disease burden in Sweden, Norway, and Iceland, compared to Denmark, points towards the success of upholding lower levels of harm with strict alcohol policies. However, sex, location and cause-specific associations indicate that the role of formal alcohol policies is highly context dependent and that other factors might influence harm as well.

## Introduction

The detrimental effects of alcohol use on morbidity and mortality are well known [1, 2]. In 2019, alcohol-attributed diseases accounted for more than 3.6% of the total disease burden globally, with the highest burden observed in high to middle-income countries [3]. In the Nordic countries, this fraction lies even higher at 5.5% [4]. Differences across societies in alcohol-attributed disease burden over time derive from a complex interplay of factors such as consumption levels, socio-cultural drinking patterns, healthcare systems, and alcohol control policies [5–7].

Attempts to mitigate the adverse consequences of alcohol on health, social, and economic outcomes have largely rested on regulations implemented by governments [8]. Higher pricing and taxation, as well as reduced physical availability, are examples of evidence-based strategies suggested to reduce alcohol consumption and, consequently, its harmful effects on health [9]. However, the composition of policies adopted at the national level varies to a large extent between countries [10] as well as the effectiveness of these policies in reducing harm for specific sexes [11, 12]. The Nordic countries (Denmark, Finland, Iceland, Norway, and Sweden) with the exception of Denmark, historically stand out as having restrictive alcohol policies, mainly through high taxation and restrictions on availability [13]. While Sweden, Finland, and Denmark have seen a liberalization of alcohol policies since the mid-1990s due to adherence to European regulations on alcohol [14], the Nordic countries still have some of the strictest alcohol-related policy frameworks in Europe [10]. At the same time, there are discernible differences between the Nordic countries in scope and number of regulations related to alcohol use, in terms of production, distribution, marketing and drunk driving limits over time, among others [15], providing a natural opportunity to investigate possible health consequences from changes in these policies.

Studies on improvements in health following alcohol policy changes in the Nordic countries are abundant. However, the majority are based on single countries and distinct policies and health outcomes [16–18] whereas only a few cross-sectional studies on country differences in alcohol policy [19], and longitudinal studies investigating the effect of policy changes on individual health behaviours [20] have been carried out. Thus, research on cross-country differences of a comprehensive set of alcohol policies in relation to alcohol-related health outcomes in the Nordic region over a longer period is still scarce.

In this study, we explored whether changes in overall alcohol policy restrictiveness were associated with overall levels of alcohol-attributed disease burden in the Nordic countries of Denmark, Iceland, Finland, Norway, and Sweden. We consequently (i) expanded the geographical scope by comparing overall changes in restrictiveness of alcohol policies in all Nordic countries, (ii) widened the temporal reach by including a period from 1990 to 2019, and (iii) included comparable measures of both fatal and non-fatal health outcomes attributed to alcohol, over time and by sex. We hypothesized that overall alcohol-attributed disease burden decreases with stricter alcohol control policies.

## Methods

We measured the overall strictness of a set of alcohol policies for each country and each individual year, making use of a modified version of the Bridging the Gap (BtG)-scale for measuring the restrictiveness of alcohol policies as developed by Karlsson and Österberg [21]. For alcohol-related health outcomes, we used the age-standardized and comparable estimates of years of life losts (YLLs), years lived with disability (YLDs), and disability adjusted life years (DALYs) attributed to alcohol from the Global Burden of Disease, Injuries and Risk Factors 2019 (GBD) Study [2].

### Alcohol policy

Observing the range of definitions of alcohol policy [22], this study focused on policies created by governments through laws and regulations, e.g. we used statutory laws as the main data source complemented with existing literature on relevant policy subgroups where data was unobtainable. Moreover, only national policies were recorded to intersect with the nationally estimated disease burden data, despite the importance of regional and local measures for reducing harm related to alcohol use [9, 23]. The data sources for statutory laws per country were collected from each country’s governmental online law libraries.

### Restrictiveness of alcohol policies

The original BtG-scale [21] builds on previous efforts to quantify alcohol policies in Europe [19, 24]. The scale is divided into seven alcohol policy subgroups: control of production, control of distribution, age limits, control of marketing, drunk driving limits, public policy, and excise taxes on alcohol. A score is given to each subgroup based on its restrictive feature through a total of 15 questions, which is then summed up into a total restrictiveness score. By way of this questionnaire a particular country can be scored with a minimum of 0 and a maximum of 40 points on a restrictiveness-scale with 40 being the most restrictive. Each policy on the scale has been assigned a weight for its effectiveness in reducing alcohol consumption and alcohol-related harm based on existing literature and expert input (Supplementary I, Table S1, see reference [21] for weighting methods).

To accommodate the original BtG-scale in this study, we adjusted the questionnaire. That is, for the subgroups ‘drunk driving’ and ‘age limits’ the original questions were not sensitive enough to record more subtle policy changes and were therefore broadened. Furthermore, recent evidence suggests a negligible effect of self-regulation in alcohol marketing [25] and we therefore omitted the question related to this topic. Last, the excise taxation subgroup was revised to account for differences in purchasing power between the countries and over time. To avoid confusion, the altered scale will from this point forward be referred to as the ‘BtG-scale Modified’ (BtG-M) (Supplementary I, Table S2).

Alcohol taxation levels, specifically excise taxes separate from any value-added tax, were collected, normalized, and indexed using a purchasing power parity (PPP)-rate. For a detailed description of this procedure see Supplementary I, Table S3.

### Burden of disease attributed to alcohol use

The burden of disease attributed to alcohol was estimated using a comparative risk assessment approach known as the population-attributable fraction, which is based on three key steps, described in detail elsewhere [3, 26]. In brief, first the effect, i.e. relative risks (RRs) of different levels of alcohol use on 23 disease outcomes (full list can be found in Supplementary II, Table S4) have been estimated from systematic reviews and meta-analyses of published literature. Second, the distribution of alcohol use in the population is estimated using data on both alcohol-stocks, unrecorded and tourist consumption, and individual level alcohol consumption. The main sources are sales data from the United Nations Food and Agriculture database, the World Health Organization’s Global Information System on Alcohol and Health databases, and surveys. Third, the burden of disease attributed to alcohol is estimated by using the distribution of alcohol use in the population, the disease-specific RRs at each level of consumption, and the burden of disease for each of the 23 health outcomes in each population.

### GBD measures

We used age-standardized and sex-specific alcohol-attributed rates of YLLs, YLDs, and DALYs for all causes per 100 000 population with 95% uncertainty interval (UI) in each Nordic country at yearly intervals between 1990 and 2019. All estimates were extracted from the GBD Global Health Data Exchange (GHDx) [27]. A distinction between delayed causes, such as cancers and diabetes, and immediate causes, such as self-harm and transport injuries, was made using time lag specifications for alcohol consumption and alcohol related harm (time to first effect) [28]. Subsequently, age-standardized DALY rates (ASDR) were created by extracting the relevant DALY numbers from GHDx and using the GBD world population to age-standardize [29] (see Supplementary II, Fig. S1, and Table S4).

### Analytical approach

We assessed the overall patterns in alcohol policy restrictiveness (BtG-M score) against alcohol attributed DALYs, YLDs, and YLLs in both sexes combined in Denmark, Finland, Iceland, Norway, and Sweden between 1990 and 2019. Additionally, we explored an association between BtG-M score and alcohol-attributed DALYs using panel data regression models, looking at both sexes combined, and separately. Initially, we applied a fixed effects regression model (Supplementary II, Table S5) to control for time-invariant differences across countries by isolating the effect of time-varying variables (BtG-M) on DALYs. Subsequently, we fitted a random effects model to examine the impact of BtG-M on ASDR while considering within- and between-country variations. The Hausman test was used to select a preferred model [30]. Following the estimation of the mixed-effects model, we generated a dot plot of the estimated random intercepts for each country along with confidence intervals. We also considered the potential delayed impact of policy changes on health outcomes by stratifying our analysis into immediate and delayed causes, applying 1-year and 5-year lags, respectively. All statistical analyses were conducted using STATA (version 18) and R.

## Results

### Alcohol policy restrictiveness

**Figure 1.**
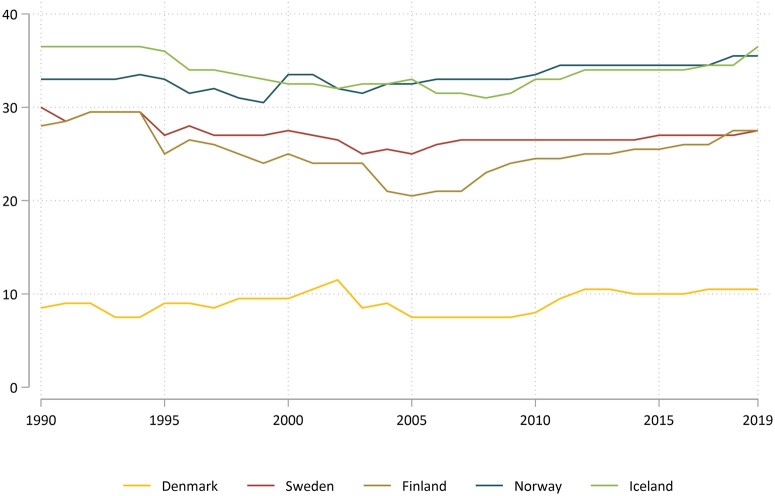
Alcohol policy restrictiveness (BtG-M score) over time by individual Nordic country.

The average BtG-M score for all countries during the study period was 25.7 (out of a possible 40) with considerable variation between countries, from 9.1 on the low end (Denmark) to 33.2 (Norway), and 33.8 (Iceland) on the high end (Fig. 1). All countries, apart from Denmark, showed an initial drop in restrictiveness around 1994-1996. After this initial period of decline, which for Sweden and Finland lasted until the early 2000s, the scores were relatively stable until 2019. Although the relative changes between 1990 and 2019 are limited, fluctuations in the 30-year period can largely be ascribed to changes in excise taxes.

### Alcohol attributed disease burden and alcohol policy restrictiveness

**Figure 2.**
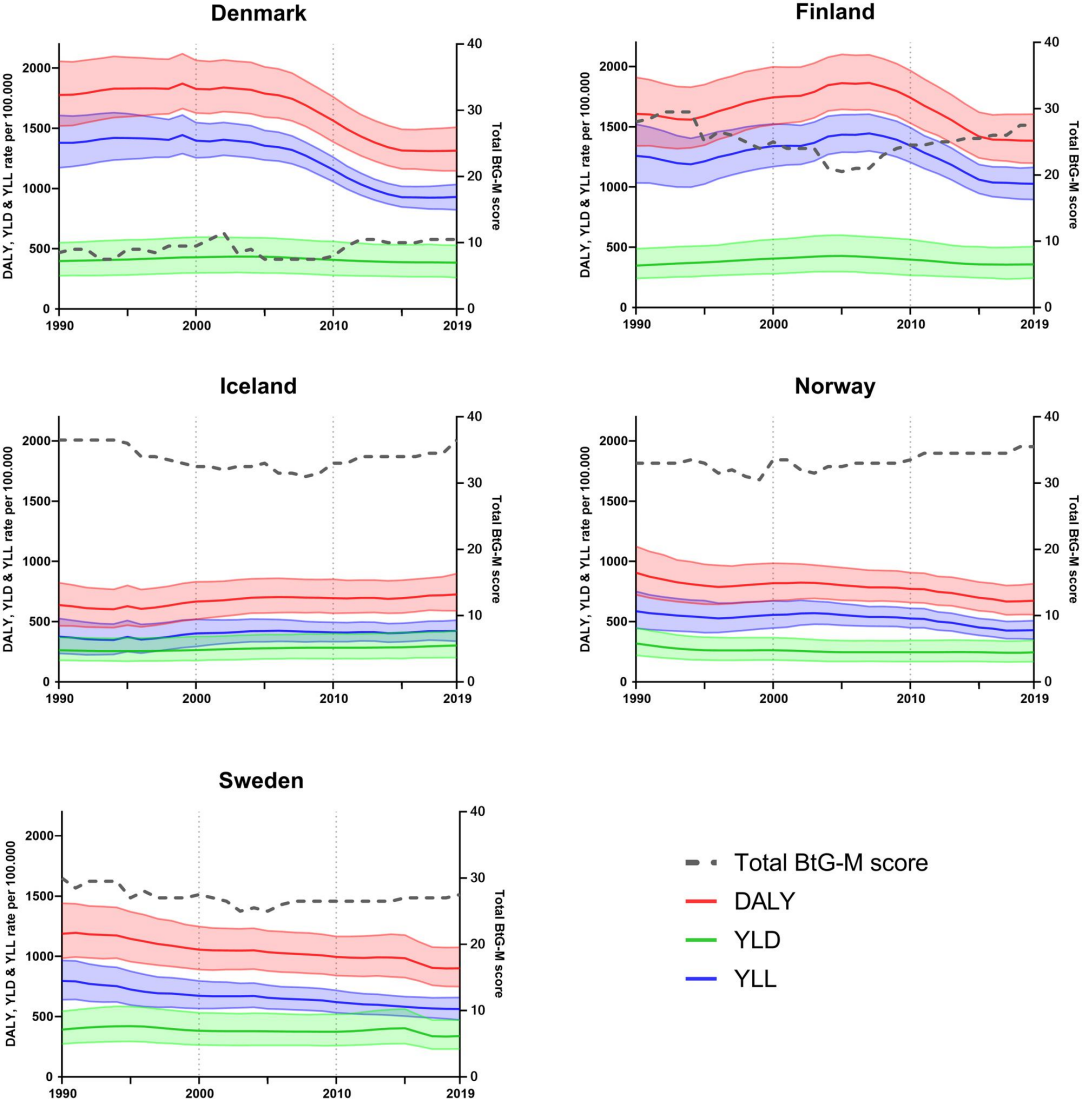
Variations in alcohol policy restrictiveness (BtG-M score) and alcohol attributed YLLs, YLDs, and DALYs (with 95% UI’s) for all Nordic countries.

Alcohol-attributed DALYs decreased in all Nordic countries with approximately 14%–26% in the past 30 years (Fig. 2), except for Iceland where an increase was detected from 638 DALYs per 100 000 in 1990 to 725 DALYs in 2019, corresponding to a 14% increase. Comparing all Nordic countries, Denmark and Finland consistently experienced a higher disease burden, despite a decrease since the mid (Denmark) and late (Finland) 2000s. The decline in alcohol-attributed DALYs in all countries but Iceland is predominantly driven by a reduction in YLLs and to a lesser extend to variations in YLDs.

Changes in disease burden and variations in the BtG-M score imply that Denmark, with the lowest overall restrictiveness over time had, together with Finland, the highest level of overall alcohol-attributed disease burden, while Iceland, Norway, and Sweden with the highest restrictiveness had the lowest burden. Finland, however, stands out with high overall restrictiveness combined with a high overall disease burden.

### Association between alcohol policy restrictiveness and alcohol-attributed disease burden

Significant negative associations were found between BtG-M scores and delayed causes, indicating that stricter alcohol policies resulted in decreased delayed causes (Table 1). Considering a 5-year lag, DALYs for delayed causes in females demonstrated a regression coefficient of −9.387 (95% CI: −11.392 to −7.381), while this was almost double for males (−21.250, 95% CI: −31.880 to −8.125).

**Table 1.** Random intercept for BtG-M scores and disease specific DALYs by sex and time lags

	No lag (*P*-value)	CI (95%)	1-year lag (*P*-value)	CI (95%)	5-year lag (*P*-value)	CI (95%)
Immediate causes						
Males	4.295 (0.729)	−19.203 to 27.794	6.364 (0.601)	−17.498 to 30.225	14.259 (0.214)	−8.208 to 36.727
Females	−2.062 (0.260)	−5.652 to 1.528	−1.734 (0.322)	−5.166 to 1.699	−0.013 (0.991)	−2.458 to 2.431
Both sexes	1.048 (0.873)	−11.811 to 13.907	2.159 (0.742)	−10.717 to 15.037	6.783 (0.256)	−4.924 to 18.490
Delayed causes						
Males	−20.891 (0.000)^a^	−27.429 to 14.354	−22.408 (0.000)^a^	−28.383 to 16.433	−21.250 (0.000)^a^	−31.880 to 8.125
Females	−10.624 (0.000)^a^	−15.033 to 6.216	−10.802 (0.000)^a^	−14.596 to 7.009	−9.387 (0.000)^a^	−11.392 to 7.381
Both sexes	−17.036 (0.000)^a^	−18.552 to 15.520	−17.257 (0.000)^a^	−18.799 to 15.715	−16.061 (0.000)^a^	−17.783 to 14.340

a *P*_bonferroni_ <0.017.

**Figure 3.**
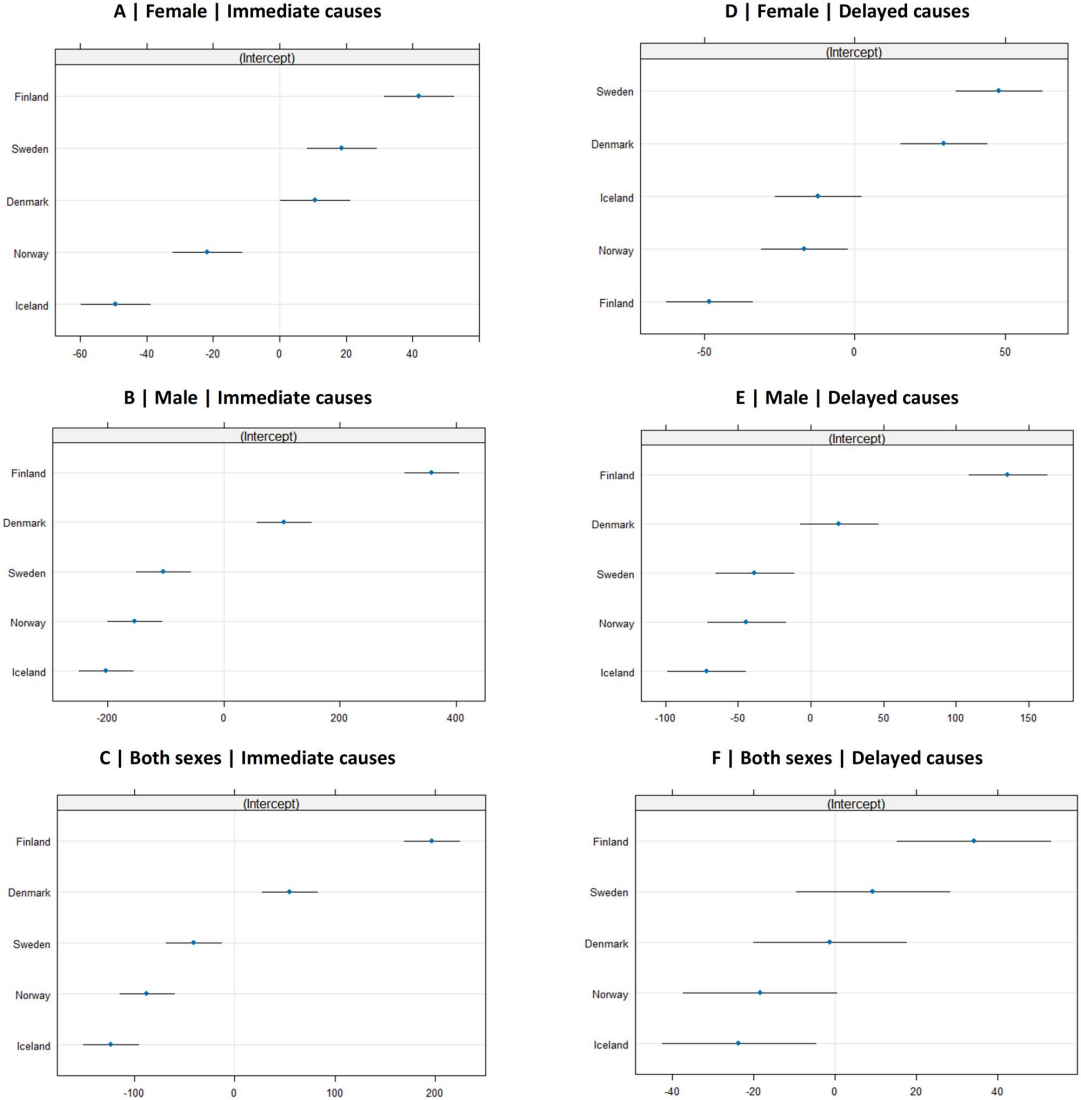
Country-specific random effects plots with estimated random intercepts and 95% CIs for immediate causes in females (A), males (B), and both sexes (C) and delayed causes for females (D), males (E), and both sexes (F).

When examining the impact of the BtG-M score on DALYs within each country, significant variations were observed for both immediate and delayed causes (Fig. 3). In nearly all combinations of sex and disease groups, Finland exhibited the greatest rise in alcohol-attributed ASDR in response to the BtG-M score, with the exception of delayed causes in females. For immediate causes, Iceland and Norway showed a negative correlation for both sexes, while Sweden displayed this trend for males only.

## Discussion

In this study, we compared changes in alcohol policy restrictiveness between 1990 and 2019 to the level of overall alcohol attributed YLLs, YLDs, and DALYs in the Nordic countries. We found that Iceland, Norway, and Sweden, with the strictest alcohol policy frameworks, had the lowest alcohol attributed disease burden throughout the observed period. Denmark confirmed this association by exhibiting the lowest BtG-M score over time, and the highest alcohol-attributed disease burden in the region. Finland, on the other hand, showed a less clear relationship with both strict policies and a relatively high disease burden compared to other Nordic countries with similar policy structures. Significant associations between BtG-M score and alcohol attributed DALYs in causes with longer time to effect were found at every time lag specification and for each sex. However, negative associations for delayed causes were only found within certain countries and for specific sexes, indicating that restrictiveness can explain the variation in the alcohol attributed disease burden in these causes only in particular contexts.

Our results on the level of alcohol policy restrictiveness for each individual Nordic country over time, e.g. where Norway was most restrictive, followed by Sweden, Finland, and Denmark correspond with the findings reported by Karlsson and Österberg using the original BtG-scale [21]. In that study, however, Iceland was not included. Several events can be identified as having shifted the overall alcohol policy score. It is well documented that concessions had to be made on certain parts of alcohol policy, such as alcohol taxation levels in an effort to limit cross-border trade [31] and the dismantling of monopolies on production of alcohol in Finland and Sweden at their introduction into the European Union (EU) [14]. Additionally, the introduction of Iceland and Norway into the European Economic Area (EEA) forced the two countries to adapt their comprehensive and restrictive alcohol policy to fit within the EEA’s more liberal and deregulated structure [32]. After this period, the BtG-M scores remained relatively stable, pointing towards the relative success of these four countries in upholding the remaining aspects of their alcohol policies.

The findings in our study on the relationship between policy and harm are generally in line with country-specific studies in Denmark [17], Finland [33], Iceland [34], Sweden [16], and Norway [35] citing in some cases an increase in harm related to more liberal alcohol policy. However, our data suggest that the burden decreased in many cases despite relatively stable, and not increasingly strict, alcohol policies. Seeing that this trend is partly driven by decreases in DALYs for immediate causes and together with the lack of associations between BtG-M score and DALYs for these causes, we find that other factors might be responsible for the drop in DALYs related to alcohol use during this period.

Denmark’s alcohol policy is, compared to the other Nordic countries, characterized by lower excise duty levels and the absence of a national monopoly on the sale of alcoholic beverages. Additionally, Denmark raised the legal purchasing age for some alcoholic beverages from 15 to 18, a policy subgroup in the BtG-M scale that has seen no change in the other four countries in the past 30 years.

The fact that Finland stands out with high alcohol-attributed disease burden irrespective of a high level of restrictiveness, is puzzling and implies that other factors might be involved. For one thing, aspects such as drinking patterns and cultural norms are known to influence consumption levels and subsequent harm [36]. Even though Finnish women did seem sensitive to increases in restrictiveness, resulting in a decrease in disease burden for delayed causes, the positive associations within Finland and Denmark, and for women in Sweden, could also imply that those who drink heavily are not affected by the policies measured, at least not those included in the BtG-M scale, or that it takes longer than our study was able to measure for formal policies to influence rigid sociocultural forces related to alcohol consumption.

Another potential explanation is the distribution of disease burden within populations. Sex differences in alcohol-attributed disease burden persist across the Nordic countries with a higher burden among males. The most distinct disparities are found in Finland, Denmark and Iceland, and trends in disease burden over time vary considerably between sexes [26]. Males seem to be more sensitive to formal alcohol policy changes compared to females, a finding consistent with literature on for instance alcohol pricing [11], although these relationships differ across distinct policies [12]. Further research is needed to understand the underlying mechanism that can explain the relation between DALYs and alcohol policy restrictiveness with regards to the impact of formal policies on different population groups such as those based on age and sex, and the effect of these stratifications for YLLs and YLDs.

There are few studies to date that explore the time lag specifications between alcohol consumption and health outcomes related to alcohol use, with varying outcomes on specific time lags for specific diseases [28, 37]. We explored a potential lag time effect between implementation of policy and assumed health effect, by linking BtG-M scores to DALYs with a 1-year (immediate)- and 5-year (delayed) lag. The significant findings for delayed causes, even without lag and with a 1-year lag, warrant further investigation. Since the time to first effect for most delayed causes is longer than 10 years [28], other factors might confound this association. Moreover, particular policies might have a different impact over time on harm depending on the disease and policy context.

### Limitations and strengths

There are limitations that need to be addressed. With the latest iteration we attempted to adjust the work of Karlsson and Österberg [21] to accommodate the policy frameworks in the Nordic region. It is possible that this scale is too crude to evaluate policy changes over time in relation to changes in disease burden. On the other hand, the use of the scale follows the comparability principle that lies at the root of the GBD study and allowed us to perform cross-country comparisons.

Second, alcohol policy is comprehensive, and its wide range of interventions can stretch to all corners of public life. With the longitudinal approach in this article and the use of a scale to capture and quantify policy restrictiveness comes failure to capture all incremental policy changes. For instance, the overhaul of Finnish alcohol retail policy in 2018 to allow low-alcoholic beverages to be sold anywhere without a license will not be picked up by our scale. Still, such policies will probably have an impact on alcohol consumption and related health outcomes. A similar issue can be observed in Norway, where the BtG-M score does not fluctuate considerably even though incremental changes in certain policies, such as an overall increase in opening hours for on- and off-premise sales, have materialized. Finally, in all countries with a monopoly framework for off-premise sales, the per capita number of sales points has increased, an indicator for availability not found within the BtG-M scale. Therefore, any restrictiveness measure and subsequent analysis must be viewed with care. However, the general trend in combination with the national alcohol-attributed disease burden can produce insight into the relationship between the two.

Third, subgroups in the BtG-M scale were challenged by the changing alcohol policy landscape and culture. For instance, even though advertisement on digital media platforms has taken up a significant part in the overall marketing of alcohol in the past decade [38], the absence of such platforms during the better part of our study period and the shift in spending on marketing between old and new platforms made comparisons more difficult. We attempted to limit this bias by only focusing on TV and Radio advertisement. Moreover, the sole focus on formal control policies has left certain (macro)economic indicators, such as change in GDP or unemployment rates that influenced alcohol consumption levels, unaccounted for.

Fourth, comparing excise taxes on alcohol across multiple years and countries proved to be challenging for two reasons. First, taxes have been levied through different mechanisms for some part of the study period in for instance Finland during the first half of the 1990s [15]. Second, taxation is often calculated using diverse grounds such as degree of alcohol of finished product or per litre of finished product. And finally, the effect on alcohol related harm is arguably limited in cases where the excise tax on alcohol did not follow per capita GDP changes, which would affect the overall affordability of alcohol.

Fifth, although attempts have been made to adjust for tourist and unrecorded consumption [3], the estimates in the GBD-study are based on consumption data that fails to consider unregistered consumption stemming from cross-border import of alcoholic beverages. The extent to which cross-border purchases affect consumption levels differs by geography and trade regulations with, and alcohol prices in neighbouring countries. For instance, the elimination of quotas for the import of alcohol in Sweden and a decrease in taxation levels for spirits in Denmark led to an increase in alcohol poisonings in southern Sweden [39], and removal of quotas in Estonia when it joined the EU has led to an increase in alcohol-related mortality in Finland, though this may have happened also as a result of a decrease of taxation levels in Finland [40].

To conclude, the Nordic countries with the strictest alcohol policy frameworks had the lowest disease burden attributed to alcohol use over the study period, except for Finland. Denmark with the lowest score on restrictiveness also had one of the highest alcohol-attributed disease burden. Changes in restrictiveness over time, however, can only partly explain the variation in alcohol attributed disease burden. Even though significant negative associations were found for delayed causes between all countries, the heterogeneity of results by sex, time lag and geography indicates that the effect of alcohol policy on alcohol attributed disease burden is complex and influenced by formal policies and socio-cultural mechanisms as well. More so, quantifying alcohol policies through a policy scale like the BtG-M, though met with challenges, is a new step in the field of longitudinal alcohol policy analysis. The use of such a scale in producing comparable insights can assist in fully understanding the variation in disease burden estimates developed within the GBD-study across countries.

## Supplementary Material

ckae195_Supplementary_Data

## Data Availability

Data on alcohol-attributed disease burden are available through the GHDx results tool at http://ghdx.healthdata.org/gbd-results-tool. Year and country specific scores for the BtG-M scale are available in the supplementary materials (Supplementary III). Key points Significant associations between restrictiveness of alcohol policies and alcohol attributed disease burden were found for some outcomes. Overall, more restrictive alcohol policies were found in countries with lower disease burdens attributed to alcohol use. Finland stands out with both a restrictive policy framework and a relatively high disease burden. Strict alcohol policies may be a crucial public health tool in reducing the disease burden from alcohol use.
