# Supplementary material for: Alcohol-attributed disease burden and formal alcohol policies in the Nordic countries (1990–2019): an analysis using the Global Burden of Disease Study 2019
Source: Eur J Public Health. 2024 Dec 2;35(1):52–9. doi: 10.1093/eurpub/ckae195 (PMC11832136; doi:10.1093/eurpub/ckae195)
Supplement: ckae195_Supplementary_Data [file ckae195_supplementary_data.zip › ckae195_Supplementary_Data/ejph-2024-06-om-0359-File006.docx]

**SUPPLEMENTARY MATERIALS**

***Supplement I***

**Table S1.** Scoring and weights of alcohol policy subgroups in the original BtG and the BtG-M Scale **2**

**Table S2.** BtG Modified scale – Questionnaire **3**

**Table S3.** BtG-M questionnaire interpretations **5**

***Supplement II***

**Table S4.** Disease groupings  **8**

**Figure S1.** Alcohol attributed age-standardized DALYs by sex and disease group for 5

Nordic countries 1990-2019  **10**

**Table S5.** Fixed effect panel data regression of alcohol attributed DALYs and BtG-M score **11**

***Supplement IV* –** Author Contributions  **12**

Supplement I

**Table S1. Scoring and weights of alcohol policy subgroups in the original BtG and the BtG-M scale^1^**

| Subgroup | Maximum points in the scale | Share of the total score (%) |
| --- | --- | --- |
| Control of production | 2 | 5⋅0 |
| Control of distribution | 10 | 25⋅0 |
| Age limits | 4 | 10⋅0 |
| Control of marketing | 3 | 7⋅5 |
| Drunk driving | 4 | 10⋅0 |
| Public Policy | 1 | 2⋅5 |
| Alcohol Taxation | 16 | 40⋅0 |
| Total | **40** | **100**⋅**0** |

**^1^** From Karlsson and Österberg [1] see also Babor et al. [2]

**Table S2: BtG Modified scale – Questionnaire (after Karlsson and Österberg’s BtG-scale [1]). Changes are highlighted in green.**

| Policy field/ subgroup | Answer/score |
| --- | --- |
| Control of production and wholesale of alcohol; State monopoly for the production or wholesale of: | Beer (.5 p) Wine (.5 p) Spirits (1 p) |
|  | **Point total -/2** |
| Control of distribution; State monopoly for off-premise sale of: | Beer (1 p) Wine (1 p) Spirits (2 p) |
| Control of distribution; No state monopoly, but restrictive license system for off-premise sales of: | Beer (.5 p) Wine (.5 p) Spirits (1 p) |
| Control of distribution; Special permanent restrictions on (*off-premise*): | Yes, sale days (1 p) Yes, sale hours (1 p) |
| Control of distribution; Other special permanent restrictions on places of sale for *off-premise* sales | Yes (1 p) |
| Control of distribution; Special permanent restrictions on (*on-premise*): | Yes, sale days (1 p) Yes, sale hours (1 p) |
| Control of distribution; Other special permanent restrictions on places of sale for *on-premise* sales | Yes (1 p) |
|  | **Point total -/10** |
| Personal Control; Legal age limit for *on-premise* sales at least: added younger age group to account for changes in earlier years | 20 for some alcoholic beverages (2 p) 18 for some alcoholic beverages (1 p) 16 for some alcoholic beverages (.5 p) |
| Personal Control; Legal age limit for *off-premise* sales at least: added younger age group to account for changes in earlier years | 20 for some alcoholic beverages (2 p) 18 for some alcoholic beverages (1 p) 16 for some alcoholic beverages (.5 p) |
|  | **Point total -/4** |
| Control of marketing, restrictions; Ban on all national alcohol advertising and sponsorship | Yes (3 p) |
| Control of marketing, restrictions; Ban on national alcohol advertisement for some alcoholic beverages | Yes (2 p) |
| Control of marketing, restrictions; Statutory control on national alcohol advertising for some alcoholic beverages | Yes (1 p) |
| ~~Control of marketing, restrictions; Voluntary code on national alcohol advertising and sponsorship~~ | ~~Yes (.5 p)~~ |
|  | **Point total -/3** |
| Social and environmental controls; Drunk driving (BAC limit) | <0.05% (4 p) 0.05% (2 p) 0.08% (1 p) |
|  | **Point total -/4** |
| Public policy; National alcohol prevention or education program | Yes (1 p) |
|  | **Point total -/1** |
| Alcohol taxation; Excise duty on strong alcoholic beverages (spirits) per hectoliter of 100% alcohol, PPP-adjusted | $1750 - $2900 (0.5)  $2901 - $3190 (1)  $3191 - $5100 (1.5)  $5101 - $5550 (2)  $5551 - $6200 (2.5)  $6201 - $7000 (3)  $7001 - $7800 (3.5)  > $7801 (4) |
| Alcohol taxation; Excise duty on intermediate products, per hectoliter of finished product, PPP-adjusted | $111 - $160 (0.5) $161 - $477 (1) $478 - $533 (1.5) $534 - $700 (2) $701 - $790 (2.5) $791 - $900 (3) $901 - $1310 (3.5) > $1311 (4) |
| Alcohol taxation; Excise duty on still wine, per hectoliter of finished product, PPP-adjusted | $71 - $100 (0.5) $101 - $232 (1) $233 - $282 (1.5) $283 - $300 (2) $301 - $415 (2.5) $416 - $550 (3) $551 - $700 (3.5) > $701 (4) |
| Alcohol taxation; Excise duty on beer, per hectoliter of finished product, PPP-adjusted | $5 - $7 (0.5) $8 - $15 (1) $16 - $22 (1.5) $23 - $28 (2) $29 - $57 (2.5) $58 - $83 (3) $84 - $172 (3.5) > $173 (4) |
|  | **Total points -/16** |
| Total score | **-/40** |

**Table S3: BtG-M questionnaire interpretations**

| **Policy subgroup** | **Interpretation** | |
| --- | --- | --- |
| **Control of production and wholesale of alcohol** | |  |
| 1. State monopoly for the production or wholesale of alcohol | Existence of a government monopoly for the production or wholesale of alcoholic beverages (beer, wine or spirits) |  |
| **Control of distribution** | |  |
| 2.1. State monopoly for off-premise sale of alcoholic beverages | Existence a government monopoly for the off-premise sale of alcoholic beverages (beer, wine or spirits) | |
| 2.2. No state monopoly, but restrictive license system for off-premise sales of alcoholic beverages | Existence of a restrictive licensing system for the off-premise sale of alcoholic beverages (beer, wine or spirits) | |
| 2.3. Special permanent restrictions on sale days and/or hours in *off-premise* sales | Restrictions of sale days and/or hours for the off-premise sales of alcoholic beverages. These imply 1) restrictions of sale hours specifically for alcoholic beverages compared to other produce sold; 2) restrictions of sale days specifically for alcoholic beverages and other than general holidays. | |
| 2.4. Other special permanent restrictions on places of sale in *off-premise* sales | Restrictions on the location of off-premise sales of alcoholic beverages, e.g., near certain functions such as schools, churches, gas stations, etc. This can also be achieved through a licencing system, in which the location of an establishment with a licence is reviewed. | |
| 2.5. Special permanent restrictions on sale days and/or hours in *on-premise* sales | Restrictions on on-premise sale days and/or hours specifically for alcoholic beverages compared to other on-premise sales. | |
| 2.6. Other special permanent restrictions on places of sale in *on-premise* sales | Restrictions on the location of on-premise sales of alcoholic beverages, e.g., near certain functions or premises (schools, churches, gas stations, work canteens, hospitals, sport clubs, etc.). This can also be achieved through a licencing system, in which the location of an establishment with a licence is reviewed. Restrictions can also imply that food or non-alcoholic beverages must be offered in addition to alcoholic beverages. | |
| **Personal Control** | |  |
| 3.1. Legal age limit for *on-premise* sales | Existence of a minimum legal age limit for the consumption and/or sale of alcoholic beverages in restaurants, bars and other on-premise locations | |
| 3.2. Legal age limit for *off-premise* sales | Existence of a minimum legal age limit for the consumption and/or sale of alcoholic beverages in alcohol sale points such as state monopolies and grocery stores | |
| **Control of marketing** | |  |
| 4.1. Ban on all national alcohol advertising and sponsorship | Due to the large timespan of the study, the focus is placed on TV/radio advertisement. For example, online, print or advertisements at sports events are therefore excluded if not in combination with a ban on TV/radio. | |
| 4.2. Ban on national alcohol advertisement for some alcoholic beverages | Ban on national advertisement for some types of beverages such as spirits or wines. | |
| 4.3. Statutory control on national alcohol advertising for some alcoholic beverages | Statutory control is interpreted as limited broadcasting times and/or target group limits, while no ban is in place. | |
| **Social and environmental controls** | |  |
| 5.1. Drunk driving (BAC limit) | Existence of drunk driving limits based on blood alcohol concentration (BAC) or equivalent. | |
| **Public policy** | |  |
| 6.1. National alcohol prevention or education program | Existence of a national-wide prevention or education programme or strategy targeting alcohol use or drug use (including alcohol) as statutory law or implemented policy. Examples: Finnish Alkoholiohjelma, Finnish national strategy for alcohol, drugs, tobacco and addictions, Swedish Alkoholkommittén, Swedish alcohol narcotics, doping, tobacco and gambling strategy. Excluded are, for example, nation-wide but relatively brief campaigns (mainly organised and financed by NGOs) if they are not part of a governmental structure or programme/strategy. | |
| **Alcohol taxation** | |  |
| 7. Excise duty on strong alcohol beverages (spirits), intermediate products, wine and beer    Data sources  Alcohol taxation levels, specifically excise taxes separate from any value added tax, were obtained from either 1) the Excise Duty Tables – Part I Alcoholic Beverages comprised and updated yearly since 1990 by the European Commission, or 2) national sources on taxation levels. Four beverage types were considered with corresponding alcohol percentages as used in the EU excise duty tables: beer (5%), wine (11%), intermediate products (18%) and spirits (22%).  Data on excise taxes was unobtainable (Iceland) or did not fit the structure of the BtG-M scale for the period 1990-1994. In some cases, excise taxes on alcohol during this period were levied based on the percentage of retail price plus a base amount (Norway) or as a percentage of profit from the alcohol-monopoly (Finland). We therefore used other sources [3] or imputed the earliest known excise tax level for each alcoholic beverage for those years that data was missing or obscured. [3]    Unit conversion  All taxes were converted to a single comparable measure per type of alcoholic beverage. These measures differ to best accommodate the different data sources by beverage group and are represented as followed; beer, wine and intermediate products, ‘per hectolitre of finished product’; and spirits, ‘per hectolitre of 100% alcohol. The before mentioned alcohol percentages were employed to calculate excise tax levels in cases where the duty was reported as degree of alcohol per volume of finished product. The assumption that 1 Degree Plato (1°P) generates approximately 0.4% alcohol by volume was used in cases where the duty was reported as °P of finished product.    Currency conversion  For countries that experienced a change in currency during the study-period, i.e. from local currency to Euro, we converted the pre-euro tax levels to euro’s using historical annual conversion rates posted by the Publication Office of the European Union (<https://data.europa.eu/data/datasets/5ym77cnmqrswdwuwli9kkw?locale=en>).    Adjustment for differences in purchasing power  To account for price changes between countries and over time Purchasing Power Parities (PPP) generated by the OECD (<https://data.oecd.org/conversion/purchasing-power-parities-ppp.htm>) were used to convert local currencies into dollars for each country-year in the study to ensure comparability across countries.    Grouping  The obtained range of PPP-adjusted excise taxes within each type of product was then used to create octiles, each representing an equal number of observations, and appropriate scores were assigned from 1-4 by steps of 0.5. | | |

Supplement II – Disease groupings

**Table S4: 23 alcohol related causes in the GBD study 2019: ICD-10 code and time-lag classification**

| ICD-10 | Disease and injuries | Time to first effect[4] | Time to full effect[4] | Disease grouping |
| --- | --- | --- | --- | --- |
| A10-A14, A15 -A18.89, A19-A19.9, B90-B90.9, K67.3, K93.0, M49.0, N74.0-N74.1, P37.0, U84.3, Z03.0, Z11.1, Z20.1, Z23.2 | Tuberculosis | Immediate | 5 years | Immediate |
| A48.1, A70, B96.0-B96.1, B97.21, B97.4-B97.6, J09-J18.2, J18.8-J18.9, J19.6-J22.9, J85.1, J91.0, P23-P23.9, U04 -U04.9, Z25.1 | Lower respiratory infections | Immediate | - | Immediate |
| I11-I11.2, I11.9 | Hypertensive heart disease | Immediate | 5 years | Immediate |
| G40-G41.9, Z82.0 | Idiopathic epilepsy | Immediate | 5 years | Immediate |
| V00-V86.99, V87.2-V87.3, V88.2-V88.3, V90-V98.8 | Transport injuries | Immediate | - | Immediate |
| D69.5-D69.59, D70.1-D70.2, D78-D78.89, D89.81-D89.813, E03.2, E06.4, E09-E09.9, E16.0, E23.1, E24.2, E27.3, E36-E36.8, E66.1, E86.02-E87.99, E89-E89.9, G21.0-G21.19, G24.0-G24.09, G25.1, G25.4, G25.6-G25.79, G62.0, G72.0, G93.7, G96.0, G96.11, G97-G97.9, H02.81-H02.819, H05.33-H05.339, H05.42-H05.53, H44.6-H44.799, H59-H59.89, H91.0-H91.09, H95-H95.9, I95.2-I95.81, I97-I97.9, J70-J70.5, J95-J95.9, K08.5-K08.59, K43-K43.9, K52.0, K62.7, K68.11, K91-K91.9, K94-K95.89, L23.3, L27.0-L27.1, L55-L55.9, L56.0-L56.1, L58-L58.9, L64.0, L76-L76.82, M10.2-M10.29, M60.2-M60.28, M87.1-M87.19, M96-M96.9, N14-N14.4, N30.4-N30.41, N46.021, N46.121, N52.2-N52.39, N65-N65.1, N99-N99.9, P93-P93.8, P96.2, P96.5, R50.2-R50.83, W00-W46.2, W49-W62.9, W64-W70.9, W73-W81.9, W83-W94.9, W97.9, W99-X06.9, X08-X44.9, X46-X58.9, Y10-Y14.9, Y16-Y19.9, Y40-Y84.9, Y88-Y88.3, Z21.0, Z42-Z43.0, Z43.8-Z43.9, Z48-Z48.9, Z51-Z51.9, Z88-Z88.9, Z92-Z94.0, Z94.6, Z94.8-Z94.9, Z96-Z96.49, Z96.6-Z97.2, Z97.8-Z99.12, Z99.3-Z99.9 | Unintentional injuries | Immediate | - | Immediate |
| X60-X64.9, X66-X84.9, Y87.0 | Self-harm | Immediate | - | Immediate |
| T74.2-T76.22, X85-Y08.9, Y87.1-Y87.2 | Interpersonal violence | Immediate | - | Immediate |
| C15-C15.9, Z85.01 | Oesophageal cancer | 10 years | 20 years | Delayed |
| C22-C22.4, C22.7-C22.9, Z85.05 | Liver cancer due to alcohol use | 10 years | 20 years | Delayed |
| C32-C32.9, Z85.21 | Larynx cancer | 10 years | 20 years | Delayed |
| C50-C50.629, C50.8-C50.929, Z12.3-Z12.39, Z80.3, Z85.3, Z86.000 | Breast cancer | 10 years | 20 years | Delayed |
| C18-C19.0, C20, C21-C21.8, Z12.1-Z12.13, Z85.03-Z85.048, Z86.010 | Colon and rectum cancer | 10 years | 20 years | Delayed |
| C00-C07, C08-C08.9, Z85.81-Z85.810 | Lip an oral cavity cancer | 10 years | 20 years | Delayed |
| C11-C11.9 | Nasopharynx cancer | 10 years | 20 years | Delayed |
| C09-C10.9, C12-C13.9 | Other pharynx cancer | 10 years | 20 years | Delayed |
| I20-I21.6, I21.9-I25.9, Z82.4-Z82.49 | Ischaemic heart disease | Immediate | 10 years | Delayed |
| G45-G46.8, I63-I63.9, I65-I66.9, I67.2-I67.848, I69.3-I69.4 | Ischaemic stroke | Immediate | 10 years | Delayed |
| I61-I62, I62.9, I69.0-I69.298 | Intracerebral haermorrhage | Immediate | 10 years | Delayed |
| I48-I48.92 | Atrial fibrillation and flutter | Immediate | 10 years | Delayed |
| I85-I85.9, I98.2, K70-K71, K71.3-K72, K72.1-K75, K75.2, K75.4-K76.2, K76.4-K77.8, R16-R18.9, Z52.6, Z94.4 | Cirrhosis and other chronic liver diseases due to alcohol use | Immediate | 20 years | Delayed |
| K85-K86.9 | Pancreatitis | Immediate | 20 years | Delayed |
| E08-E08.11, E08.3-E08.9, E10-E10.11, E10.3-E11.1, E11.3-E12.1, E12.3-E13.11, E13.3-E14.1, E14.3-E14.9, R73-R73.9, Z13.1, Z83.3 | Diabetes mellitus | Immediate | 10 years | Delayed |

***References***

1. Karlsson, T. and E. Österberg, *Scaling alcohol control policies across Europe.* Drugs : education, prevention & policy, 2007. **14**(6): p. 499-511.

2. Babor, T., et al., *Alcohol: No Ordinary Commodity – a summary of the second edition*. 2010. p. 769.

3. Horverak, Ø. and E. Österberg, *Alkoholprisene i Norden.* Nordisk Alkoholtisdkrift (Nordic Alcohol Studies), 1991. **8**(3): p. 127-142.

4. Holmes, J., et al., *The temporal relationship between per capita alcohol consumption and harm: A systematic review of time lag specifications in aggregate time series analyses.* Drug and alcohol dependence, 2011. **123**(1): p. 7-14.

**Figure S1: Alcohol attributed age-standardized DALYs by sex and disease group for 5 Nordic countries 1990-2019**

**Table S5: Fixed effect panel data regression of alcohol attributed DALYs and BtG-M score**

|  | **Fixed effects** | | | | |  |
| --- | --- | --- | --- | --- | --- | --- |
|  | **NO LAG**  **(*P-value)*** | ***CI (95%)*** | **1-YEAR LAG (*P-value)*** | ***CI (95%)*** | **5-YEAR LAG**  **(*P-value)*** | ***CI (95%)*** |
| **Immediate causes** |  | | | | | |
| Males | 8.233 (0.582) | -30.005 - 46.470 | 10.802 (0.467) | -26.547 - 48.151 | 19.259 (0.160) | -11.753 - 50.271 |
| Females | -.852 (0.771) | -8.439 - 6.735 | -.470 (0.857) | -7.249 - 6.310 | 1.318 (0.250) | -1.4024 - 4.038 |
| Both sexes | 3.526 (0.671) | -17.838 - 24.889 | 4.905 (0.542) | -15.526 - 25.336 | 9.828 (0.158) | -5.892 - 25.548 |
| **Delayed causes** |  | | | | | |
| Males | -18.333 (0.038)* | -35.047 - -1.619 | -21.074 (0.020)* | -36.807 - -5.341 | -20.003 (0.009)** | -31.882 - -8.125 |
| Females | -9.104 (0.065) | -19.134 - .926 | -9.483 (0.044)* | -18.549 - -.416 | -6.846 (0.032)* | -12.734 - -.958 |
| Both sexes | -13.692 (0.048)* | -27.229 - -.154 | -15.314 (0.027)* | -27.851 - -2.777 | -13.636 (0.014)** | -22.803 - -4.469 |

**p* < 0.05

***p_bonferroni_  < 0.017*

Supplement IV - Author contributions (in alphabetical order)

*Conceptualization*

Emilie E Agardh, Anna-Karin Danielsson, Thomas G Karlsson, Lode van der Velde, Peter Wennberg.

*Data curation*

Hassan Abolhassani, Emilie E Agardh, Peter Allebeck, Jennifer L Baker, Anna-Karin Danielsson, David Edvardsson, Seyed-Mohammad Fereshtehnejad, Pär Flodin, Mika Gissler, Rasmus J Havmoeller, Thomas G Karlsson, Ali Kiadaliri, Adnan Kisa, Sezer Kisa, Ann Kristin Skrindo Knudsen, John J McGrath, Atte Meretoja, Tuomo J Meretoja, Christopher J L Murray, Che Henry Ngwa, Gavin Pereira, Inga Dora Sigfusdottir, Jens Christoffer Skogen, Thomas Clement Truelsen, Lode van der Velde, Tommi Juhani, Vasankari, and Peter Wennberg.

*Formal Analysis*

Emilie E Agardh, Jennifer L Baker, Anna-Karin Danielsson, Pär Flodin, Thomas G Karlsson

Adnan Kisa, Christopher J L Murray, Anastasia Månsson, Ahmed Nabil Shabaan, Inga Dora

Sigfusdottir, Lode van der Velde, and Peter Wennberg.

*Funding Acquisition*

Emilie E Agardh.

*Investigation*

Emilie E Agardh, Ahmed Nabil Shabaan, Lode van der Velde.

*Methodology*

Emilie E Agardh, Jennifer L Baker, Anna-Karin Danielsson, Pär Flodin, Thomas G Karlsson

Adnan Kisa, Christopher J L Murray, Anastasia Månsson, Ahmed Nabil Shabaan, Inga Dora

Sigfusdottir, Lode van der Velde, and Peter Wennberg.

*Project Administration*

Emilie E Agardh, Lode van der Velde.

*Software*

Ahmed Nabil Shabaan, Lode van der Velde.

*Validation*

Hassan Abolhassani, Emilie E Agardh, Peter Allebeck, Johan Ärnlöv, Ashokan Arumugam, Muhammad Asaduzzaman, Jennifer L Baker, Koustuv Dalal, Anna-Karin Danielsson, Keshab Deuba, David Edvardsson, Terje Andreas Eikemo, Pär Flodin, Mika Gissler, Rasmus J Havmoeller, Simon I Hay, Knud Juel, Thomas G Karlsson, Joonas H Kauppila, Ali Kiadaliri, Adnan Kisa, Sezer Kisa, Mika Kivimäki, Ann Kristin Skrindo Knudsen, Tea Lallukka, Anders O Larsson, Mall Leinsalu, John J McGrath, Atte Meretoja, Tuomo J Meretoja, Junmei Miao Jonasson, Christopher J L Murray, Subas Neupane, Che Henry Ngwa, Maja Pasovic, Gavin Pereira, Ahmed Nabil Shabaan, Rahman Shiri, Inga Dora Sigfusdottir, Jens Christoffer Skogen, Indra de Soysa, Thomas Clement Truelsen, Lode van der Velde, and Peter Wennberg.

*Visualization*

Ahmed Nabil Shabaan, Lode van der Velde

*Writing – Original Draft Preparation*

Emilie E Agardh, Peter Allebeck, Anna-Karin Danielsson, Terje Andreas Eikemo, Pär Flodin, Mika Gissler, Thomas G Karlsson, Ann Kristin Skrindo Knudsen, John J Mcgrath, Anastasia Månsson, Maja Pasovic, Ahmed Nabil Shabaan, Inga Dora Sigfusdottir, Rannveig Sigurvinsdottir, Jens Christoffer Skogen, Lode van der Velde, Peter Wennberg.

*Writing – Review & Editing*

Hassan Abolhassani, Emilie E Agardh, Peter Allebeck, Johan Ärnlöv, Ashokan Arumugam, Muhammad Asaduzzaman, Jennifer L Baker, Koustuv Dalal, Anna-Karin Danielsson, Keshab Deuba, David Edvardsson, Terje Andreas Eikemo, Pär Flodin, Mika Gissler, Rasmus J Havmoeller, Simon I Hay, Knud Juel, Thomas G Karlsson, Joonas H Kauppila, Ali Kiadaliri, Adnan Kisa, Sezer Kisa, Mika Kivimäki, Ann Kristin Skrindo Knudsen, Tea Lallukka, Anders O Larsson, Mall Leinsalu, John J McGrath, Atte Meretoja, Tuomo J Meretoja, Junmei Miao Jonasson, Christopher J L Murray, Subas Neupane, Che Henry Ngwa, Maja Pasovic, Gavin Pereira, Ahmed Nabil Shabaan, Rahman Shiri, Inga Dora Sigfusdottir, Jens Christoffer Skogen, Indra de Soysa, Thomas Clement Truelsen, Lode van der Velde,and Peter Wennberg.
